# PERCEPTIONS OF BRAIN HEALTH AND INTEREST IN PARTICIPATING IN BRAIN HEALTH RESEARCH AMONG ADULTS AGE 50 TO 64

**DOI:** 10.1093/geroni/igz038.1525

**Published:** 2019-11-08

**Authors:** Erica Solway, Donovan Maust, Matthias Kirch, Dianne Singer, Jeffrey Kullgren, Preeti Malani

**Affiliations:** 1 Institute for Healthcare Policy and Innovation, University of Michigan, Ann Arbor, Michigan, United States; 2 University of Michigan, Ann Arbor, Michigan, United States; 3 University of Michigan Medical School, Ann Arbor, Michigan, United States

## Abstract

Evidence suggests it may be possible to reduce the risk of developing dementia during midlife. The University of Michigan National Poll on Healthy Aging (NPHA), a nationally representative online survey, sought to determine to what extent adults age 50 to 64 anticipate and worry about developing dementia, are taking steps to prevent dementia, and are likely to participate in dementia-related research. Nearly 50% of poll respondents (n=1,025) perceived themselves as being somewhat or very likely to develop dementia. Worry about developing dementia was higher among respondents who had a family member with dementia (66.3% vs. 31.8%; Pearson’s Chi squared, p<0.001) and those who had been a caregiver of a person with dementia (65.2% v. 38.9%; Pearson’s Chi squared p<0.001). Only 5% of respondents had discussed preventing dementia with their doctor. In contrast, many respondents endorsed pursuing a variety of strategies to help maintain their memory. For example, 55% did crossword puzzles or other brain games; more than 30% reported taking fish oil or omega-3 supplements. Finally, 44% of respondents said they were likely to participate in studies to test a new medicine to prevent dementia and to test a new treatment for dementia. According to this NPHA, while many adults age 50 to 64 in the U.S. are worried about developing dementia, fewer are willing to participate in research to prevent or treat dementia. The low percentage who discussed dementia prevention with their doctor is concerning, particularly because many report using non-evidence-based prevention strategies such as dietary supplements.

